# Dr. Harry Cumley

**Published:** 1892-02

**Authors:** 


### DEATH OF DR. HARRY CRUMLEY.

It is with the profoundest sense of regret that we feel called upon to make mention of the death of Dr. Harry Crumley, of Chattanooga, who died in that city January 25th, of pneumonia, after a brief illness.

Although young in years, he was looked upon as one of the foremost physicians of the city in which he lived, and held the chairs of Anatomy, and Diseases of the Mind and Nervous System, in the Chattanooga Medical College. For two years previous to his settlement in Chattanooga, he was physician to the Dayton and Columbus Insane Asylums.

In him the profession will recognize the loss of brilliant and gifted genius, and the laity, a skilled physician and benefactor.

